# *In Vitro* Antioxidant Effects of *Aloe* *barbadensis* Miller Extracts and the Potential Role of These Extracts as Antidiabetic and Antilipidemic Agents on Streptozotocin-Induced Type 2 Diabetic Model Rats

**DOI:** 10.3390/molecules171112851

**Published:** 2012-11-01

**Authors:** Mohammed Moniruzzaman, Begum Rokeya, Sohel Ahmed, Amrita Bhowmik, Md. Ibrahim Khalil, Siew Hua Gan

**Affiliations:** 1 Biomedical Research Group, Department of Pharmacology, Bangladesh Institute of Research and Rehabilitation in Diabetes, Endocrine and Metabolic Disorders (BIRDEM), Dhaka, 1000, Bangladesh; Email: b_rokeya@yahoo.com (B.R.); amritabhwmk@gmail.com (A.B.); 2 Department of Pharmacology, School of Medical Sciences, Universiti Sains Malaysia, Kubang Kerian 16150, Kelantan, Malaysia; Email: mibrahim12@yahoo.com; 3 Department of Biochemistry and Molecular Biology, Jahangirnagar University, Dhaka, 1342, Bangladesh; Email: drsahmed_bmbju@yahoo.com; 4 Human Genome Centre, School of Medical Sciences, Universiti Sains Malaysia, Kubang Kerian 16150, Kelantan, Malaysia; Email: shgan@kck.usm.my

**Keywords:** *Aloe vera*, streptozotocin-induced diabetic rats, antioxidants, antidiabetic, DPPH, FRAP

## Abstract

In this study, the total phenolic and flavonoid contents, the 2,2-diphenyl-1-picryl hydrazyl (DPPH) radical scavenging ability and the ferric reducing power (FRAP) of *Aloe vera* were measured to determine the antioxidant activity of this species. The *in vivo* antidiabetic effects of the plant were also investigated using streptozotocin-induced type 2 diabetic model rats that were divided into five groups based on the treatment received: (1) water (WC); (2) glibenclamide; (3) concentrated gel extract (Gel-C); (4) ethanol (80%) gel extract (Gel-Et); and (5) ethanol (80%) skin extract of *Aloe vera* (Skin-Et). Skin-Et, which contained the highest level of total phenolics (62.37 ± 1.34 mg_gallic acid_/kg) and flavonoids (20.83 ± 0.77 mg/kg), exhibited the highest scavenging activity (85.01 ± 0.52%) and the greatest reducing power (185.98 ± 0.41 µM), indicating that the skin contained the highest level of antioxidants. The oral consumption of Gel-Et for 4 weeks a caused significant reduction in the fasting serum glucose levels of the rats. The rats in the Gel-C-, Gel-Et- and Skin-Et-treated groups experienced a reduction in their total cholesterol levels by 11%, 17% and 25%, respectively and a reduction in their LDL cholesterol levels by 45%, 3% and 69%, respectively. The *in vivo* experimental antioxidant parameter MDA is strongly correlated with the *in vitro* antioxidant parameters of flavonoids and polyphenols, namely the DPPH and FRAP values (r = 0.94, 0.92, 0.93, 0.90), thus confirming the antioxidant potential of the *Aloe vera* extracts.

## 1. Introduction

Type 2 diabetes mellitus poses a major global health threat, especially in developed and developing countries [1] and is now considered a worldwide epidemic [2]. The prevalence and complications of type 2 diabetes are increasing daily. The use of conventional drugs to treat metabolic disorders and the pathological consequences of diabetes further increases the complications because of the side effects and high costs of these drugs. Therefore, there is a need to develop alternative strategies for diabetes therapy. Natural products are useful alternatives because these compounds are believed to have fewer side effects.

Many herbs, spices and other plant materials have been used to treat diabetes. A total of more than 400 species were reported to display hypoglycemic effects, but few of these species have actually been investigated [3]*.* To date, 90 plants have been screened for hypoglycemic properties at the Bangladesh Institute of Research and Rehabilitation in Diabetes, Endocrine and Metabolic Disorders (BIRDEM) and at institutions in neighboring countries. These plants include *Trigonella foenum graecum*, *Allium cepa*, *Hemidesmus indicus*, *Syzigium cumini*, *Murraya koenigii* and *Aloe vera*.

*Aloe vera* has been used for many centuries for its curative and therapeutic properties [4,5], its antibacterial, antifungal and antiviral activities [6] and of its ability to treat hyperlipidemia and psoriasis symptoms [7]. Moreover, *Aloe vera* reduces the intestinal absorption of water, helping to increase the occurrence of bowel movements. As a result, this plant is widely used as a laxative [8]. Topical and oral uses of *Aloe vera* gel have been shown to increase the collagen content in experimental dermal wounds in rats [9,10]. Additionally, histological examinations showed early epithelialization in the *Aloe vera* gel-treated skin areas [11]. Both the *Aloe vera* gel and skin are purported to have antidiabetic and cytoprotective activities, which may help ameliorate complications from diabetes and cardiovascular diseases. *Aloe vera* has also been reported to have antioxidant activities [12,13,14].

In diabetes mellitus, chronic hyperglycemia produces multiple biochemical abnormalities and diabetes-induced oxidative stress can play a role in the symptoms and progression of the disease [15,16]. It has been reported that oxidative stress is also one of the major causes of pathophysiological conditions during diabetes. Moreover, persistent hyperglycemia may lead to an exaggerated production of free radicals, especially reactive oxygen species (ROS), in tissues due to glucose oxidation and protein glycosylation [17,18,19]. Diabetes is also associated with hyperlipidemia. To reduce the risk of late complications and negative outcomes of diabetes mellitus such as renal failure, blindness and limb amputation, the control of both blood glucose levels an lipid levels is important [20,21].

The hypoglycemic effects of *Aloe vera* have been investigated by various researchers [22,23]. Ethanolic extracts of *Aloe vera* administered to Wistar albino rats with high blood glucose levels resulted in a significant decrease in the plasma glucose levels of the rats [24]. Furthermore, when *Aloe arborescens* from the *Aloe* genus was administered to mice in a powdered form, increases in the blood sugar levels of mice that received a basal diet were significantly suppressed compared with those of the control group [25]. In this study, we investigated the antidiabetic and antioxidant effects of different *Aloe vera* extracts in streptozotocin (STZ)-induced type 2 diabetic model rats.

## 2. Results

The approximate yields for Gel-C, Gel-Et and Skin-Et were 0.5%, 0.59% and 4.0% (w/w), respectively.

### 2.1. Aloe vera Extracts Properties

*Aloe vera* skin exhibited the highest concentrations of polyphenols and flavonoids. The lowest polyphenol concentration was observed in the Gel-C extract, whereas the Gel-Et extract exhibited the lowest flavonoid content (Table 1). Among the different *Aloe vera* extracts tested, the lowest scavenging activity was observed for the Gel-Et extract, whereas the highest activity was observed for the Skin-Et extract. The highest FRAP value was also measured for the Skin-Et extract, whereas the Gel-Et extract was observed to have the lowest FRAP value (Table 1).

**Table 1:** Total phenolic and flavonoid contents, DPPH scavenging activities (%) and FRAP values of the different extracts of *Aloe vera*.

Type of *Aloe vera* Extract	Total Phenolic Compounds (Mean ± SD) (mg_gallic acid_/kg)	Flavonoids (Mean ± SD) (mg_catechein_/kg)	DPPH (Mean ± SD) (RSA %)	FRAP (Man ± SD) (µM Fe(II)/kg)
Gel-C	8.69 ± 1.18 ^b^	7.43 ± 0.03 ^c^	11.93 ± 0.58 ^b^	59.12 ± 0.18 ^b^
Gel-Et	11.38 ± 0.94 ^b^	5.43 ± 0.38 ^b^	6.56 ± 0.32 ^c^	26.51 ± 1.15 ^c^
Skin-Et	62.37 ± 1.34 ^a^	20.83 ± 0.77 ^a^	85.01 ± 0.52 ^a^	185.98 ± 0.41 ^a^

Data represent the mean ± S.D. of four independent determinations; A comparison between the groups was performed using a one-way ANOVA and a post Hoc Dunnett T3 test; Means within the same column that are labeled with different letters (superscripts) are significantly different (*p* *<* 0.05).

### 2.2. Effects of Different Treatments on the Fasting Serum Glucose Levels of Type 2 Diabetic Rats

**Figure 1:**
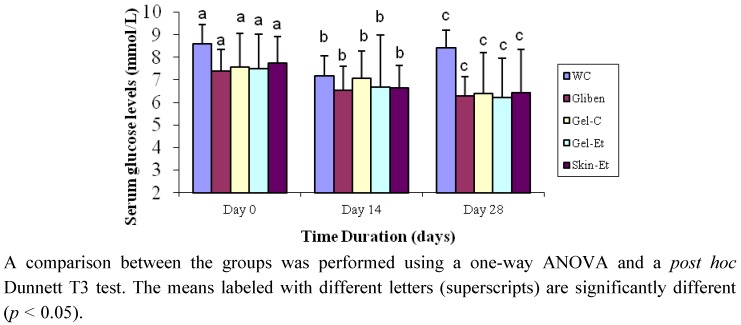
Effects of different treatments on the fasting serum glucose levels of type 2 rats. The results are expressed as the mean ± SD. Water control, WC; glibenclamide control, Gliben; *Aloe vera* concentrated gel extract, Gel-C; *Aloe vera* gel ethanol extract, Gel-Et; *Aloe vera* skin ethanol extract, Skin-Et.

There was no significant difference in the fasting serum glucose (FSG) levels of the rats between the five experimental groups at baseline (Figure 1). After the oral administration of the five different treatments over 28 days, the FSG levels decreased in the rats of all of the groups except those of the WC group. However, this decrease was not statistically significant. Type 2 diabetic rats that were treated with the ethanolic extract of *Aloe vera* gel exhibited a significant (*p* = 0.047) decrease in their FSG levels on days 14 and 28 of the experiment compared with the baseline values. As expected, glibenclamide also resulted in significant (*p* = 0.026) amelioration of the FSG levels on day 28. However, on day 28, the FSG levels increased significantly (*p* = 0.022) at the conclusion of the treatment.

### 2.3. Effects of Different Treatments on the Serum Insulin Levels and Glycogen Contents of Type 2 Diabetic Rats

There was a 20% increase in the serum insulin levels on day 28 compared with the baseline values for the rats that received Gel-Et. The serum insulin levels remained almost unchanged on day 28 for the glibenclamide-treated group compared with the baseline values. However, for the other three groups, a reduction was observed in the serum insulin levels. No change was observed in the hepatic glycogen contents among the tested groups on day 28.

### 2.4. Effects of Different Treatments on the Serum Lipid Profile and the HDL and LDL Levels of Type 2 Diabetic Model Rats

**Figure 2:**
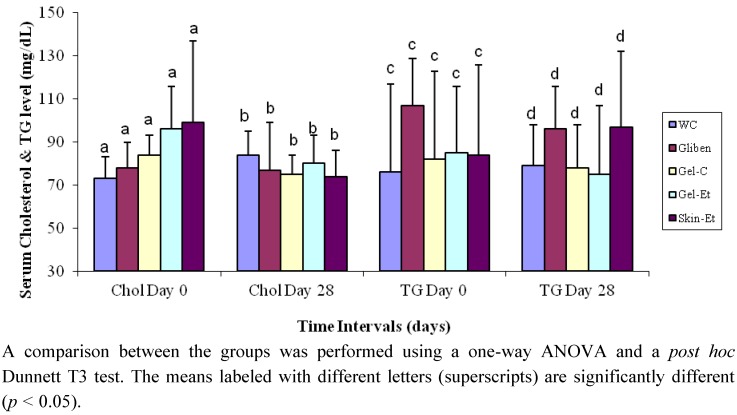
Effects of different treatments on the serum lipid profile of type 2 diabetic model rats. Chol = Cholesterol; TG = Triglycerides.

On day 28, the rats in the Gel-C, Gel-Et and Skin-Et groups exhibited decreases in their serum cholesterol levels by 11%, 17% and 25%, respectively (Figure 2), whereas the rats that received glibenclamide exhibited only a 1% decrease. In contrast, the rats in the WC group exhibited an increase of 15% in their total serum cholesterol levels. In the case of serum triglycerides, type 2 model rats from the WC and Skin-Et groups exhibited increased levels (by approximately 4% and 15%, respectively). The rest of the groups experienced a decrease in triglycerides (10% for the glibenclamide-treated rats, 5% for the Gel-C-treated rats and 12% for the Gel-Et-treated rats).

**Figure 3:**
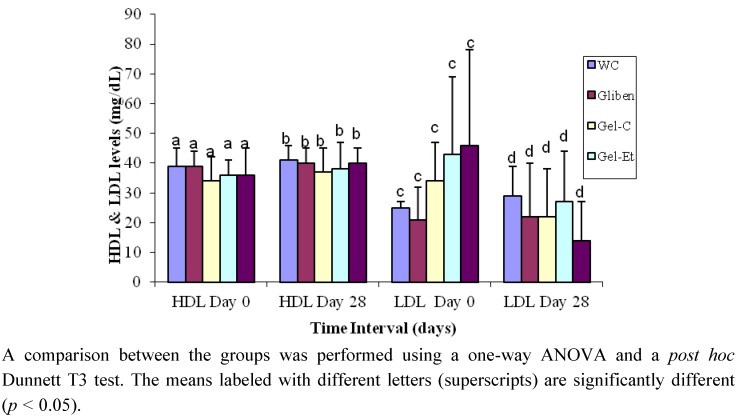
Effects of different treatments on the serum HDL and LDL levels of type 2 diabetic model rats.

The glibenclamide-treated rats exhibited decreases in their serum HDL and LDL levels by 3% and 9%, respectively (Figure 3). The HDL cholesterol levels increased by 14%, 5% and 5% in the Gel-C, Gel-Et and Skin-Et groups, respectively. A reduction of approximately 45%, 3% and 69% in the LDL levels were observed when the rats received Gel-C, Gel-Et and Skin-Et treatments, respectively, indicating that the *Aloe vera* preparations are able to reduce atherogenic LDL cholesterol levels. Furthermore, the protective effects of *Aloe vera* were more pronounced on the atherogenic LDL cholesterol levels than on the HDL cholesterol levels in the type 2 diabetes model rats.

### 2.5. Effects of Different Treatments on the MDA and GSH Levels of Erythrocytes from Type 2 Diabetes Model Rats

**Figure 4:**
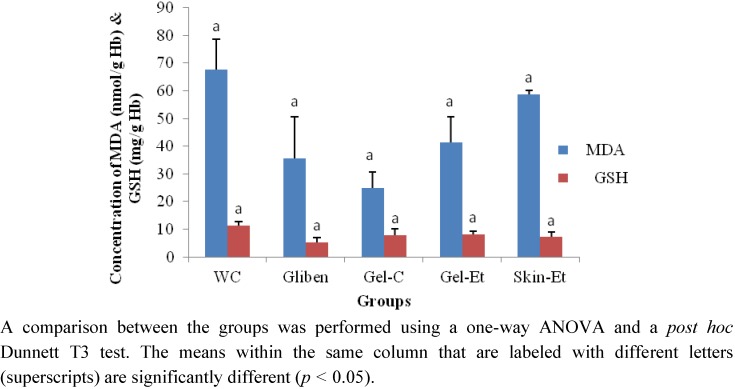
Effects of different treatments on the erythrocyte MDA and GSH levels in type 2 diabetic model rats.

On day 28, the erythrocyte MDA levels were lower in the rats of the glibenclamide, Gel-C and Gel-Et groups compared with those of the rats in the WC group (Figure 4). However, the rats treated with Skin-Et exhibited higher MDA levels. In terms of the reduced GSH levels, the observed levels were all lower than that of the control group.

### 2.6. Correlation

Several significant correlations were observed between the biochemical and antioxidant parameters obtained in our study. Strong correlations were found among the levels of polyphenols, flavonoids, DPPH and FRAP, indicating that these parameters are good indicators of the antioxidant activities of *Aloe vera* (Table 2). The correlations between the phenolic compounds and DPPH and FRAP were r = 0.986 and 0.962, respectively. The most significant correlation was observed between the flavonoid content and the DPPH values, for which r = 0.997. Moreover, the *in vivo* experimental antioxidant parameter MDA is also strongly correlated with the *in vitro* antioxidant parameters (levels of flavonoids and polyphenols) and biochemical assay results (DPPH and FRAP), indicating the antioxidant potency of the investigated *Aloe vera*.

**Table 2:** Correlation matrix showing the interrelation among the levels of polyphenols, flavonoids, DPPH, FRAP, GSH and MDA.

	Polyphenols	Flavonoids	DPPH	FRAP	GSH	MDA
**Polyphenols**	1	0.982 *	0.986 *	0.962 *	0.716 *	0.944 *
**Flavonoids**	0.982 *	1	0.997 *	0.995 *	0.629	0.920 *
**DPPH**	0.986 *	0 .997 *	1	0.991 *	0.663	0.991 *
**FRAP**	0.962 *	0.995 *	0 .991 *	1	0.610	0.900 *
**GSH**	0.716 *	0.629	0 .663	0.610	1	0.621
**MDA**	0 .944 *	0.920 *	0 .939 *	0.900 *	0.621	1

* Correlation is significant at the 0.01 level (2-tailed).

## 3. Discussion

The total phenolic and flavonoid contents and the results of the DPPH and FRAP assays confirmed that *Aloe vera* is a good source of antioxidants. Although this concept has also been demonstrated in previous studies [26,27,28,29], our study is the first to show that various preparations of *Aloe vera* have different antioxidant potentials and that the skin exhibits the highest antioxidant properties.

The total polyphenol contents from the different *Aloe vera* preparations were investigated using the modified Folin-Ciocalteu assay, which is sensitive to phenol and polyphenol entities and other electron-donating antioxidants, such as ascorbic acid and vitamin E. The total phenolic composition of *Aloe vera* in our study is similar to that determined in previous studies [26,27,28,29]. By preventing hyperglycemia-induced oxidative stress and the associated pancreatic β-cell destruction, *Aloe vera* plant extracts increase insulin secretion via the pancreas, thus ameliorating diabetes-associated hyperglycemia and dyslipidemia [30,31,32]. This effect is attributed to the various polyphenols present in the *Aloe vera* extract that lower glucose uptake and prevent hyperglycemia [33,34].

In our study, the total flavonoid contents of the different *Aloe vera* preparations were also determined. Flavonoids were present in lower amounts than were the phenolics, as previously reported [26,27,28,29]. However, our results demonstrate that among the three different extracts of the *Aloe vera* samples, the *Aloe vera* skin exhibited the highest content of both phenolic and flavonoid compounds (62.37 ± 1.34 mg/kg and 20.83 ± 0.77 g/kg, respectively), indicating that the skin must be used when processing *Aloe vera* as a potential antioxidant agent.

Significant differences were observed among the *Aloe vera* samples in terms of their scavenging abilities, which were expressed as the percentage of inhibition of the DPPH radical (Table 1). The DPPH radical scavenging test is one of the fastest tests available for investigating the overall hydrogen/electron-donating activity of single antioxidants and health-promoting dietary antioxidant supplements. To evaluate the radical-scavenging potential of honey solutions, the DPPH radical reaction is frequently used because the antioxidant potential of *Aloe vera* is directly related to the phenolic and flavonoid content of the extract [27,29,35,36]. The markedly higher radical scavenging capacity exhibited by different *Aloe vera* preparations is most likely a result of the high content of antioxidant species. Among the three different extracts of *Aloe vera*, the skin extract exhibited the highest value of ferric reducing power, indicating the potential use of the skin as an antioxidant agent.

Because hyperglycemia-induced oxidative stress is purported to be involved in the etiology for diabetes [27,29,35,36], natural herbs containing antioxidants may be useful for ameliorating diabetes. Furthermore, *Aloe vera* has traditionally been used as a tonic for treating diabetes. To date, there have been various animal studies in which the anti-diabetic properties of a variety of other *Aloe* species have been investigated and *Aloe vera* is by far the best described species. Interventions involving various extracts, including the use of 80% aqueous ethanol extracts of these *Aloe* species, have also been shown to alleviate diabetes.

In our study, the *in vitro* findings were further confirmed by conducting *in vivo* tests in diabetic model rats. The serum glucose levels were measured at baseline on day 14 and day 28. On day 14, although there was a decrease in the serum glucose levels in the rats treated with all of the *Aloe vera* extracts and in the glibenclamide-treated rats, the results were not statistically significant. However, after day 28, the serum glucose levels were significantly lower compared both with those at baseline and with those obtained on day 14. Although a reduction in the fasting blood glucose levels was also observed in the rats treated with the concentrated gel and ethanol gel extracts of *Aloe vera*, the rats treated with the skin ethanol extract exhibited the highest reduction, indicating the potential of this extract to serve as a hypoglycemic agent. Our results are similar to the findings reported by Rajasekaran *et al*. [37] and Noor *et al*. [38] who indicated that an *Aloe vera* gel extract was effective in lowering hyperglycemia levels in STZ-induced diabetic rats although an earlier report indicated that the administration of an *Aloe vera* extract to rabbits led to hyperglycemia [39]. In another study, a significant improvement in the serum glucose levels in diabetic rats was observed in animals treated with an *Aloe vera* juice filtrate compared with the results obtained for non-diabetic control animals [40].

The hypoglycemic effect of plant extracts generally depends on the degree of β-cell destruction. Thus, the hypoglycemic effect of *Aloe vera* may be due to a peripheral effect of the *Aloe vera* extract. The elevated levels of reduced liver glycogen in type 2 diabetic rats as a result of treatment with both gel and skin extracts of *Aloe vera* suggests an improvement in liver glycogenesis. Glycogen is the primary intracellular storable form of glucose and its levels in various tissues are a direct reflection of insulin activity because insulin promotes the activity of intracellular glycogen synthase and inhibits glycogen phosphorylase [41,42].

It is also possible that the reduction in the blood glucose levels was due to a reduction in food intake. However, when the body weights of the control and treated rats were compared, there was no noticeable change in the body weight between the different groups of rats, indicating that diet is not a factor that affects blood glucose levels.

In our study, the chronic administration of *Aloe vera* extracts over 28 days caused a decrease in the serum cholesterol and triglyceride levels relative to the levels observed for the glibenclamide-treated (positive control) and water-treated (control) groups (Figure 2). The serum HDL cholesterol levels increased and the LDL cholesterol levels decreased in all rat groups relative to the baseline (Figure 3), indicating that *Aloe vera* extracts exert hypolipidemic effects on type 2 diabetic model rats. Type 2 diabetes is also associated with a marked imbalance in lipid metabolism [43], and diabetic dyslipidemia is characterized by a low level of HDL cholesterol and elevated levels of total cholesterol, triglycerides and LDL cholesterol. The association of hyperglycemia with an alteration of lipid parameters presents a major risk for cardiovascular complications in diabetes. In addition to poor glycemic control, treatment of hyperlipidemia also results in significant micro- and macrovascular diseases in individuals with type 2 diabetes [44]. Hence, an improvement in the lipid profiles will reduce diabetic complications. The results of the present investigation also supported the previous study conducted by Rajasekaran *et al*. [20] who confirmed that the oral administration of an *Aloe vera* gel extract for 21 days resulted in a significant reduction in the plasma cholesterol and triacylglycerol levels. Moreover, in another study, an eight-week administration of processed *Aloe vera* gel to mice caused a significant decrease in the levels of serum triacylglycerols [22].

The rats that were treated with crude and ethanol gel extracts and glibenclamide exhibited lower MDA levels compared with those in the WC-treated group (Figure 4), indicating the inhibitory role of these extracts on the oxidative damage of erythrocytes. However, the GSH levels involved in the protection against reactive oxygen species [45,46] did not increase as expected following the *Aloe vera* treatment, although the antioxidant properties of *Aloe vera* have been reported [12,14]. In previous reports, the antioxidant properties of *Aloe vera* were determined by measuring the oxidative metabolism on human neutrophils and by characterizing and purifying glutathione peroxidase and superoxide dismutase. In our *in vivo* study, we did not observe any significant effects of different preparations of *Aloe vera* on STZ-induced diabetic rats. This result may be due to the severe damage of pancreatic β cells and other complications in the model rats used [47,48].

## 4. Experimental

### 4.1. Plant Material and Sample Preparation

In June 2009, *Aloe vera* leaves (20 kg) were collected from commercially available sources in Dhaka city, Bangladesh. The botanical name of the investigated *Aloe vera* species is *Aloe barbadensis* Miller. The fleshy leaves were washed several times with distilled water and dried at room temperature so the outer skin of the *Aloe vera* was devoid of water droplets. The stiff and spiny edges from both sides of the *Aloe vera* skin were then removed using knives. The green outer skin and the gel (*i.e.*, the pulpy portion inside the leaves) were carefully separated and the respective portions were individually weighed (Scientech SA 210, Boulder, CO, USA) before being chopped and blended using a commercially available blender.

*Preparation of concentrated Aloe vera gel*: After blending, the *Aloe vera* gel was concentrated under a reduced pressure using a rotary evaporator, freeze-dried (at −55 °C) and transferred to a reagent bottle to be stored in a freezer (at −8 °C).

*Preparation of ethanol (80%) Aloe vera gel and skin*: The blended *Aloe vera* gel and skin were extracted using ethanol (80%) as a solvent. Following the extraction, both the gel and the outer green skin extract were concentrated under reduced pressure using a rotary evaporator. The semi-dried ethanolic extract was then freeze-dried (at −55 °C) and transferred to a reagent bottle to be stored in a freezer (at −8 °C).

For the *in vitro* experiments, the Gel-C (10 g), Gel-Et (10 g) and Skin-et samples were dissolved in 80% ethanol at a 1:1 (w/v) ratio. The mixture was sonicated for 10 min and shaken using a Bigger Bill-120 Shaker (Thermolyne, Dubuque, IA, USA) for 1 h. The solvent was collected after centrifugation at 3,000 × *g* for 10 min. This process was repeated twice to ensure the total extraction of all compounds from the lyophilized extracts. The supernatants were pooled and evaporated to dryness under reduced pressure using a rotary evaporator. The residue was subsequently stored in dry sterilized containers at −20 °C until further use.

### 4.2. Chemicals and Reagents

1,1-Diphenyl-2-picrylhydrazyl (DPPH), 2,4,6-tripyridyl-s-triazine (TPTZ), gallic acid and Folin and Ciocalteu’s phenol reagent were purchased from Sigma-Aldrich (St. Louis, MO, USA). Sodium carbonate (Na_2_CO_3_), aluminum chloride (AlCl_3_), sodium nitrite (NaNO_2_), ferrous sulfate heptahydrate (FeSO_4_·7H_2_O), ferric chloride hexahydrate (FeCl_3_·6H_2_O) and sodium hydroxide (NaOH) were purchased from Merck (Darmstadt, Germany).

### 4.3. Animals and Study Design

Adult Long-Evans female rats (180–220 g) were used in this study. The animals were bred at the BIRDEM animal house in Dhaka, Bangladesh, at a constant room temperature of 22 ± 5 °C at a humidity between 40% and 70%. The animals received a natural 12 h day-night cycle. The rats were provided with a standard laboratory pellet diet and water *ad libitum*. Type 2 diabetes was induced by a single intraperitoneal injection of STZ in citrate buffer (10 mL) at a dose of 90 mg/kg to the rat pups (48 hours old, average weight 7 g) as described by Bonner-Weir *et al*. [49]. The experiments were performed three months after the STZ injection. The type 2 diabetic model rats were selected for the experiment after conducting an oral glucose tolerance test (OGTT) and only the diabetic model rats with blood glucose levels of 8–12 mmol/L under fasting conditions were selected for the experiments. Thirty-four female rats were divided into the following five groups: group 1 (n = 7), water-treated control rats; group 2 (n = 7), glibenclamide-treated rats; group 3 (n = 6), Gel-Et-treated rats; group 4 (n = 7), Gel-C-treated rats; and group 5 (n = 7), Skin-Et-treated rats. All treatments were given for 28 consecutive days.

To evaluate the anti-diabetic activity of each extract, the extracts were administered orally for 28 days at a dose of 1.25 g/kg. Only a single daily dose was administered to the rats. For all of the pharmacological studies, the drug glibenclamide was administered orally to the rats at a dose of 5 mg/10 mL (9.9 mL H_2_O + 0.1 mL Twin 20) per kg of Type 2 model rat [50]. The animals in the control groups received 10 mL water per kg of body weight.

Blood samples (1 mL) were collected by cutting the tail tips of the rats at baseline as well as on day 14. At the end of day 28, the rats were sacrificed and blood samples (5 mL) were collected again to measure several different parameters. These parameters included the fasting blood glucose levels at baseline and on day 14 and the glucose, insulin, creatinine, triglycerides (TG), total cholesterol (TC), low-density lipoprotein (LDL), high-density lipoprotein (HDL), malondialdehyde (MDA) and reduced glutathione (GSH) levels at baseline and on day 28. The glucose, insulin, creatinine, TG, TC, LDL and HDL levels were measured using the serum, whereas the MDA and GSH levels were measured from the RBC homolysate. The body weights of the individual rats were also measured in seven-day intervals.

### 4.4. Determination of Total Phenolic Compounds

The concentrations of phenolic compounds from the *Aloe vera* samples were estimated using a modified spectrophotometric Folin-Ciocalteu method [51]. Briefly, the different extracts of *Aloe vera* (Gel-C, Gel-Et and Skin-Et) (1 mL) were mixed with Folin and Ciocalteu’s phenol reagent (1 mL). After 3 min, a 10% Na_2_CO_3_ solution (1 mL) was added to the mixture which was diluted to 10 mL with distilled water. The reaction remained in the dark for 90 min, and then the absorbance was measured at 725 nm using a T 80 UV/VIS spectrophotometer (ChromoTek GmbH, Planegg-Martinsried, Germany). Gallic acid was used to calculate the standard curve (20, 40, 60, 80 and 100 µg/mL, r^2^ = 0.996). The concentration of phenolic compounds was measured in triplicate. The results are reported as the mean ± standard deviation and expressed as milligrams of gallic acid equivalents (GAEs) per kg of *Aloe vera* extracts.

### 4.5. Determination of Total Flavonoids

The total flavonoid content (TF) of each *Aloe vera* sample was determined using a previously developed colorimetric assay [52]. The *Aloe vera* extract (1 mL) was mixed with distilled water (4 mL). At baseline, NaNO_2_ (0.3 mL, 5% w/v) was added. After 5 min, AlCl_3_ (0.3 mL, 10% w/v) was added and this step was followed by the addition of NaOH (2 mL, 1 M) 6 min later. Immediately, the volume was increased to 10 mL by the addition of distilled water (2.4 mL). The mixture was vigorously shaken to ensure adequate mixing and the absorbance was measured at 510 nm. A calibration curve was prepared using a standard solution of catechin (20, 40, 60, 80 and 100 µg/mL, r^2^ = 0.997). The results were also expressed as mg of catechin equivalents (CEQ) per kg of honey.

### 4.6. DPPH Free Radical-Scavenging Activity

The antioxidant properties of each *Aloe vera* extract were also studied by evaluating the free radical-scavenging activity of the DPPH radical. This activity was measured using a previously reported method [53]. Briefly, each *Aloe vera* extract (1 mL) was mixed with methanolic solution containing DPPH radicals (2.7 mL, 0.024 mg/mL). The mixture was vigorously shaken and left to stand for 60 min in the dark until the absorbance of the solution remained unchanged. The degree of reduction of the DPPH radical was determined by measuring the absorbance of the mixture at 517 nm [54]. The radical-scavenging activity (RSA) was calculated as the percentage of DPPH discoloration using the following equation:

% RSA = [(A_DPPH_−A_S_)/A_DPPH_] × 100

where A_S_ is the absorbance of the solution when the sample extract has been added at a particular level and A_DPPH_ is the absorbance of the DPPH solution.

### 4.7. FRAP Assay

The ferric reducing antioxidant activity was assayed using a previously described method [55]. The absorbance of the *Aloe vera* was measured at 593 nm using a T80 UV/VIS spectrophotometer (ChromoTek GmbH, Planegg-Martinsried, Germany). These determinations were performed in quadruplicate and the results are expressed as FRAP values [µM Fe(II) in the *Aloe vera* solution (10%)].

### 4.8. Estimation of Serum Glucose

The glucose oxidase (GOD-PAP) test was performed using the method published by Trinder [56].

### 4.9. Estimation of Serum Insulin

The serum insulin levels were estimated using the rat insulin ELISA (enzyme-linked immunosorbant assay) kit (Crystal Chem Inc., Downers Grove, IL, USA) according to the method described by Kratzsch *et al*. [57].

### 4.10. Measurement of Glycogen from Rat Liver

The glycogen levels in the rat liver were estimated using the anthrone-sulfuric acid method [58].

### 4.11. Estimation of Total Serum Cholesterol

The serum cholesterol levels of the rats were determined after enzymatic hydrolysis and oxidation. The indicator quinoneimine was prepared from hydrogen peroxide and 4-aminoantipyrine in the presence of phenol and peroxidase [59]*.*

### 4.12. Estimation of Serum Triglyceride (TG)

The serum triglyceride levels of the rats were measured using an enzymatic colorimetric glycerol-3-phosphate oxidase phenol aminophenazone (GPO-PAP) method using an Automatic Analyzer (Hitachi 704, Hitachi Ltd., Tokyo, Japan) and reagents from Randox Laboratories Ltd. (Crumlin, UK) [60].

### 4.13. Estimation of MDA and GSH Levels

#### 4.13.1. Preparation of Erythrocyte Hemolysate

Approximately 2 mL of blood from each rat was collected by cervical decapitation into individual test tubes containing 3 units of heparin. After 5 min, the heparinized blood was centrifuged at room temperature at 1,200 × *g* for 5 min, forming a white fibrin clot in the upper layer of the plasma. Large forceps were used to squeeze the clot to leave a stringy, white fibrin mass. The RBCs that remained were lysed with five volumes of distilled deionized water [61].

#### 4.13.2. Estimation of Hemoglobin

The hemoglobin concentration in the rat RBCs was estimated using a reagent kit (Human, Wiesbaden, Germany) for a photometric colorimetric test using the cyanmethemoglobin method described by Van Kampen and Zijlstra [62].

#### 4.13.3. Estimation of Erythrocyte MDA Levels

The degree of lipid peroxidation was estimated by measuring the MDA levels (which served as a lipid peroxidation index) using the thiobarbituric acid method reported by Srour *et al*. [63].

#### 4.13.4. Estimation of the Reduced Glutathione (GSH) Levels in Erythrocytes

The GSH concentration in the erythrocytes was measured using the method published by Ellman [64].

### 4.14. Statistical Analysis

The data from our experiments were analyzed using the Statistical Package for Social Science (SPSS) Software for Windows version 12 (SPSS Inc., Chicago, IL, USA). All of the data were expressed as the mean ± SD or median (range) whenever appropriate. The statistical analysis of the results was performed using Student’s t-test (paired and unpaired), the Mann Whitney U test and an ANOVA (analysis of variance) followed by the Bonferroni and Dunnett post hoc tests. The limit of significance was set at *p* < 0.05.

## 5. Conclusions

The ethanolic skin extracts of *Aloe vera* were observed to contain the highest total phenolic and flavonoid contents. Furthermore, these extracts exhibited high DPPH scavenging activities and FRAP values, indicating the potential of this plant to be used as an antioxidant. The concentrated and ethanolic *Aloe vera* gel extracts also exhibited significant antioxidant properties, albeit to a lesser degree. The *Aloe vera* extracts also have the potential to be used as hypoglycemic agents, especially the skin extract. Additionally, although the result is not statistically significant, the ethanol skin extract exhibited potent hypolipedemic effects by decreasing the serum cholesterol and LDL levels by 25% and 69%, respectively, while also increasing the HDL levels. Further studies are required to isolate the active principle(s) and elucidate the mechanism of the antioxidant and antidiabetic effects of the *Aloe vera* plant to explore the role of this plant as a potential antidiabetic agent.
